# The dual nature of DNA damage response in obesity and bariatric surgery-induced weight loss

**DOI:** 10.1038/s41419-024-06922-0

**Published:** 2024-09-11

**Authors:** David Israel Escobar Marcillo, Valeria Guglielmi, Grete Francesca Privitera, Michele Signore, Valeria Simonelli, Federico Manganello, Ambra Dell’Orso, Serena Laterza, Eleonora Parlanti, Alfredo Pulvirenti, Francesca Marcon, Ester Siniscalchi, Veronica Fertitta, Egidio Iorio, Rosaria Varì, Lorenza Nisticò, Mahara Valverde, Paolo Sbraccia, Eugenia Dogliotti, Paola Fortini

**Affiliations:** 1 Dept of Environment and Health, ISS, Viale Regina Elena 299, 00161 Roma, Italy; 2 Internal Medicine Unit and Obesity Center, University Hospital Policlinico Tor Vergata, Rome, Italy; 3 Department of Clinical and Experimental Medicine, Bioinformatics Unit, University of Catania, Catania, Italy; 4 Core Facilities, ISS, Viale Regina Elena 299, 00161 Roma, Italy; 5 High Resolution NMR Unit-Core Facilities, ISS, Viale Regina Elena, 299, 00161 Roma, Italy; 6 Center for Gender-Specific Medicine, ISS, Viale Regina Elena 299, 00161 Rome, Italy; 7 Centre for Behavioral Sciences and Mental Health, ISS, Viale Regina Elena 299, 00161 Roma, Italy; 8 Instituto de Investigaciones Biomédicas, Universidad Nacional Autónoma de México, C.U. C.P, 04510 CDMX, México

**Keywords:** Senescence, Predictive markers

## Abstract

This novel study applies targeted functional proteomics to examine tissues and cells obtained from a cohort of individuals with severe obesity who underwent bariatric surgery (BS), using a Reverse-Phase Protein Array (RPPA). In obese individuals, visceral adipose tissue (VAT), but not subcutaneous adipose tissue (SAT), shows activation of DNA damage response (DDR) markers including ATM, ATR, histone H2AX, KAP1, Chk1, and Chk2, alongside senescence markers p16 and p21. Additionally, stress-responsive metabolic markers, such as survivin, mTOR, and PFKFB3, are specifically elevated in VAT, suggesting both cellular stress and metabolic dysregulation. Conversely, peripheral blood mononuclear cells (PBMCs), while exhibiting elevated mTOR and JNK levels, did not present significant changes in DDR or senescence markers. Following BS, unexpected increases in phosphorylated ATM, ATR, and KAP1 levels, but not in Chk1 and Chk2 nor in senescence markers, were observed. This was accompanied by heightened levels of survivin and mTOR, along with improvement in markers of mitochondrial quality and health. This suggests that, following BS, pro-survival pathways involved in cellular adaptation to various stressors and metabolic alterations are activated in circulating PBMCs. Moreover, our findings demonstrate that the DDR has a dual nature. In the case of VAT from individuals with obesity, chronic DDR proves to be harmful, as it is associated with senescence and chronic inflammation. Conversely, after BS, the activation of DDR proteins in PBMCs is associated with a beneficial survival response. This response is characterized by metabolic redesign and improved mitochondrial biogenesis and functionality. This study reveals physiological changes associated with obesity and BS that may aid theragnostic approaches.

## Introduction

Obesity is a multifactorial disease resulting from the interaction between genetic and environmental factors and nowadays has reached epidemic proportions [1].

The obese phenotype increases the risk of developing metabolic syndrome (MS) [2], which in turn raises the susceptibility to various non-communicable diseases, including certain cancers [3]. Additionally, obesity is associated with the onset of several complications such as type 2 diabetes (T2DM), cardiovascular disease (CVD), and stroke [4].

Currently, bariatric surgery (BS) is the most effective treatment for severe obesity, ensuring a considerable and long-term reduction of body weight, as well as the remission of the most common obesity-related morbidities [5, 6]. However, negative outcomes related to BS have also been identified [7, 8].

Numerous studies have addressed the impact of obesity and therapeutic surgery on health outcomes by examining adipose tissue and circulating peripheral blood mononuclear cells (PBMCs). These studies have consistently documented the presence in these tissues of inflammation, metabolic derangements, and oxidative stress, all of which are recognized as contributors to DNA damage. For example, an increase of both nuclear phosphorylated histone H2AX foci and micronuclei in PBMCs of obese/overweight individuals, compared to normal weight (NW) controls, was reported in an Italian cohort of children in association with increased levels of circulating pro-inflammatory cytokines [9]. An increase of DNA breaks and micronuclei frequency was reported in blood and lymphocytes of patients with metabolic syndrome versus healthy controls [10]. Additionally, in line with the beneficial health effects of BS-induced weight loss, more recent studies have reported a significant reduction in DNA breaks in PBMCs from patients with obesity one year after surgery. Unexpectedly, no reduction in oxidative damage to DNA was found [11, 12]. While the connection between inflammation, oxidative stress and DNA damage in the context of obesity starts to emerge, there still remains a substantial knowledge gap concerning their association and the underlying mechanisms.

Another unanswered question pertains to the connection between severe obesity, physiological aging and DNA damage accumulation. A growing body of evidence indicates that obesity and aging share several phenotypic features, such as progressive white adipose tissue dysfunction, systemic chronic inflammation, and multi-organs alterations (adipaging) [13, 14]. DNA damage accumulation and chronic activation of DDR lead to cellular senescence characterized by the release of matrix metalloproteases, growth factors, pro-inflammatory cytokines and chemokines, a process known as senescence-associated secretory phenotype (SASP) [15, 16].

In recent years, there has been an increasing amount of evidence highlighting the relationship between excessive nutrient intake and mitochondrial dysfunction. Indeed, an overload of calories induces oxidative stress, leading to mitochondrial dysfunction that, in turn, intensifies the production of ROS, creating a harmful cycle that contributes to the development of chronic inflammation [17]. Furthermore, a functional association between telomeres, oxidative stress and mitochondria is increasingly recognized [18] highlighting their pivotal role in aging and metabolic syndrome [19].

In this study, we tested the hypothesis that increased production of metabolic byproducts, generated by chronic excessive caloric intake, causes irreparable DNA damage accumulation and chronic DDR activation, leading to cellular senescence, local and systemic chronic inflammation and metabolic derangement in target tissues. Additionally, we aimed to examine whether therapeutic surgery could potentially reverse these effects. To this end, we enrolled a cohort of subjects affected by severe obesity who underwent BS and collected both pre-surgery visceral (VAT) and subcutaneous adipose tissue (SAT) biopsies, as well as PBMCs samples, before and after BS, with one-year follow-up assessment. Proteomics represents a valuable approach to identify possible biomarkers for diagnosis and targeted therapy. Untargeted, mass spectrometry analysis allows broad proteomic analysis but requires high amounts of input material for targeted enrichment of specific sub-fractions of the proteome such as post-translational modifications. Therefore, we opted for a targeted, antibody-based approach and used the Reverse Phase Protein microArrays (RPPA) technology, that allowed us to quantify relative levels of key phosphorylated, i.e. activated, DDR players starting from a few tens of micrograms of protein extracts. The RPPA was performed in both VAT and SAT biopsies to evaluate the expression pattern of markers of DDR, senescence, and obesity-related metabolic changes. Furthermore, to gain insights into the functional implications of the proteomics profile, mitochondrial health and telomere dynamics were characterized.

## Results

RPPA-based proteomics on preselected proteins was conducted in both adipose tissue and PBMCs from 36 subjects affected by severe obesity who had well-documented clinical and biochemical profiles and were eligible for BS (Supplementary information table S1). To obtain reference values for our biomarkers, samples from 16 NW subjects were used [8]. In the case of PBMCs, the analysis was extended to include samples collected during the post-BS time, up to a 1-year follow-up period.

### Chronic DDR is associated with senescence in VAT of subjects with obesity

**Fig. 1:**
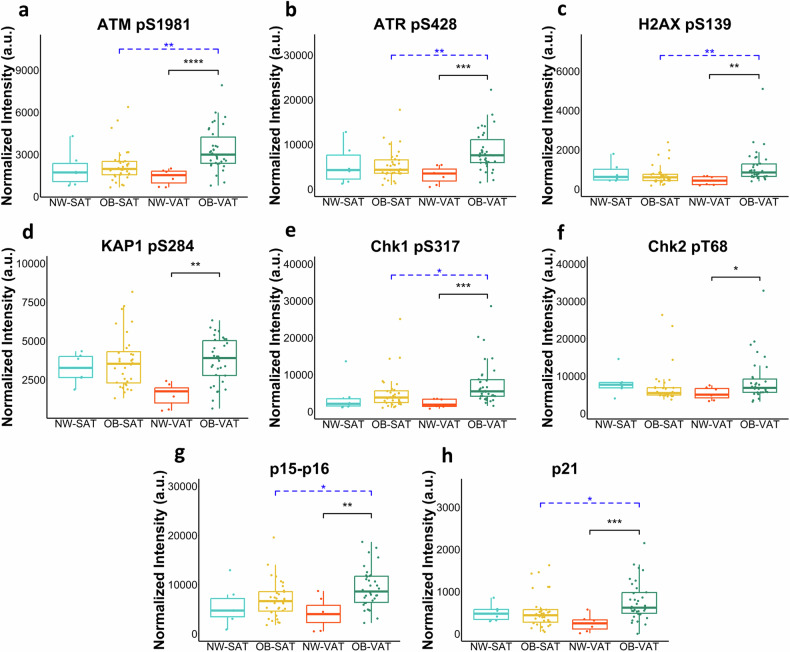
DDR and senescence markers are activated in adipose tissue of obese individuals. Box plots resulting from comparison of RPPA profiles of biopsies of subcutaneous adipose tissue (SAT) and visceral adipose tissue (VAT) of severely obese patients (OB-SAT and OB-VAT) and normal-weight (NW) controls (**a**–**h**). The plots represent the distribution of RPPA intensity values (median ± SD). Statistical comparisons were performed with Wilcoxon rank sum test as non-parametric test and t-test as parametric test for not-paired samples (black brackets). Paired samples were compared using Wilcoxon signed-rank test as non-parametric test and t-test as parametric test (blue brackets). Statistical significance is coded with an asterisk according to the level of significance (*p < 0.05, **p < 0.01, ***p < 0.001, ****p < 0.0001).

The RPPA profile of DDR and senescence markers in VAT and SAT biopsies from our group of subjects with obesity was compared with that of NW individuals. As shown in Fig. 1, the phosphorylation levels of ATM (Fig. 1a) and ATR (Fig. 1b), as well as those of their recognized downstream effectors, histone H2AX (Fig. 1c) and KAP1 (Fig. 1d), were significantly higher in the VAT biopsies of subjects affected by obesity compared to NW individuals. Additional investigations into the DDR cascade revealed that the cell cycle checkpoint kinases, Chk1 (Fig. 1e) and Chk2 (Fig. 1f), were also activated. Phosphorylation levels of p15-16 (Fig. 1g) and p21 (Fig. 1h), two readouts of senescence, were found to be higher in the VAT tissue samples from individuals with obesity compared to controls. In contrast, no significant differences in the levels of these biomarkers were found when comparing SAT specimens from obese subjects to control individuals (Fig. 1a–h). In addition, in individuals with obesity, the activation level of most of these proteins was higher in VAT than in SAT specimens (Fig. 1).

Our findings demonstrate the concurrent activation of DDR and senescence markers in the VAT, but not in the SAT, in our cohort of individuals with obesity. It is worth noting that our previous findings have shown elevated levels of inflammatory and oxidative markers in the plasma of these subjects (Fig S1) [8].

This aligns with the activation of SASP in the omental tissue of individuals with obesity who exhibit chronic low-grade inflammation.

### Stress-responsive metabolic markers are specifically increased in omental adipose tissue of individuals with severe obesity

**Fig. 2:**
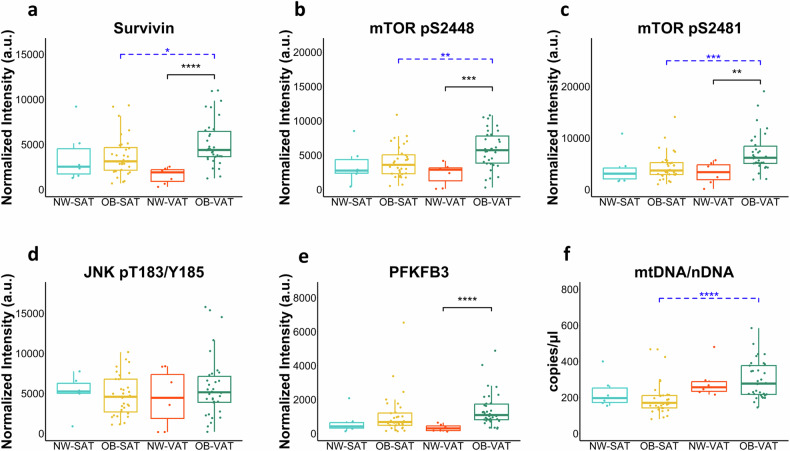
Metabolic markers of obesity are elevated in adipose tissue of obese individuals. Box plots resulting from comparison of the RPPA profiles of biopsies of subcutaneous adipose tissue (SAT) and visceral adipose tissue (VAT) of severely obese patients (OB-SAT and OB-VAT) and normal-weight (NW) controls (**a-e**). The plots represent the distribution of RPPA intensity values (median ± SD). The mtDNA/nDNA ratio was measured by digital droplet PCR (copy/µl) (**f**). Statistical comparisons were performed with Wilcoxon rank sum test as non-parametric test and t-test as parametric test for not-paired samples (black brackets). Paired samples were compared using Wilcoxon signed-rank test as non-parametric test and t-test as parametric test (blue brackets). Statistical significance is coded with an asterisk according to the level of significance (*p < 0.05, **p < 0.01, ***p < 0.001, ****p < 0.0001).

The levels of obesity-related metabolic markers, for which there is growing evidence of an association with DDR, were also monitored (Fig. 2). A significant increase in the levels of the anti-apoptotic protein survivin was observed in VAT, but not in SAT biopsies, compared to controls. VAT biopsies exhibited higher survivin expression levels compared to SAT biopsies (Fig. 2a). mTOR, a major nutritional and stress sensor, exerts its activity in two major complexes, mTORC1 and mTORC2. The first complex contains mTOR mainly phosphorylated at Ser2448, while mTORC2 is characterized by phosphorylation at Ser2481 [20]. Both mTOR Ser2448 (Fig. 2b) and mTOR Ser2481 (Fig. 2c) levels were significantly increased only in VAT samples of subjects affected by obesity when compared to controls, and higher activation of mTOR was also observed in VAT versus SAT biopsies of individuals with obesity. Although JNK and obesity-related inflammation are closely connected, the levels of JNK (Fig. 2d) in both VAT and SAT were not significantly different from controls. Nonetheless, a few VAT samples showed relatively high levels of JNK. The levels of PFKFB3 (Fig. 2e), the major regulator of glycolysis, showed a significant increase in VAT but not in SAT from patients with obesity versus adipose tissues from NW individuals.

Consistent with the elevated stress and metabolic demand of VAT in individuals with obesity, a higher mitochondrial content, as quantified by the mitochondrial-to-nuclear DNA (mtDNA/nDNA) ratio, was measured in VAT versus SAT (Fig. 2f).

Overall, these findings indicate that VAT is the critical target of metabolic changes associated with obesity-related stressors.

### A specific cell response is activated in PBMCs of individuals affected by severe obesity after bariatric surgery

**Fig. 3:**
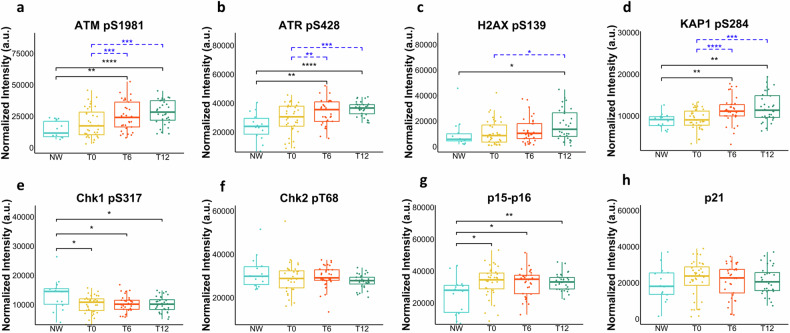
DDR markers are activated in PBMCs after bariatric surgery. Box plots resulting from the comparison of the RPPA profiles of peripheral blood mononuclear cells (PBMCs) of severely obese patients before (T0) and six (T6), and 12 (T12) months after bariatric surgery and normal-weight (NW) controls. **a**–**h** The plots represent the distribution of RPPA intensity values (mean ± SD), and statistical comparisons between OB and NW subjects were performed using the Wilcoxon rank sum test for non-normal data and t-test for normal distributed data. For the comparison at the different times, we used two tests for paired samples: Wilcoxon signed-rank test as non-parametric test and t-test as parametric test. Statistical significance is coded with an asterisk according to the level of significance (*p < 0.05, **p < 0.01, ***p < 0.001, ****p < 0.0001).

To determine if the proteomics profile of VAT in individuals with obesity could be reflected systemically in the bloodstream, we examined the same markers in PBMCs. The convenient accessibility and non-invasive nature of this biological matrix enabled us to conduct RPPA profiling not only at the enrolment time but also at various time points up to one year after BS. As shown in Fig. 3, unlike the observations in adipose tissue, there were no significant differences in the levels of phosphorylated DDR and senescence markers at baseline (T0) when comparing PBMCs from obese subjects with NW individuals. However, there was a tendency towards elevated levels of phosphorylated ATM (pATM) (Fig. 3a), ATR (pATR) (Fig. 3b), H2AX (γ-H2AX) (Fig. 3c) and KAP1 (pKAP1) (Fig. 3d) in subjects with obesity. Following surgery (T6 and T12), an unexpected increase of the levels of pATM (Fig. 3a), pATR (Fig. 3b) and pKAP1 (Fig. 3d) was observed showing an upward trend as a function of the post-surgery time while the levels of γ-H2AX (Fig. 3c) showed a significant increase only at T12. It is worth noting that no activation of Chk1 (Fig. 3e) nor of Chk2 (Fig. 3f) was detected. At baseline, PBMCs showed increased levels of p15-p16 (Fig. 3g) compared to NW controls while no changes were observed in post-surgery time points. The levels of p21 (Fig. 3h) remained unchanged both at baseline when compared to NW controls as well as at post-surgery time points.

**Fig. 4:**
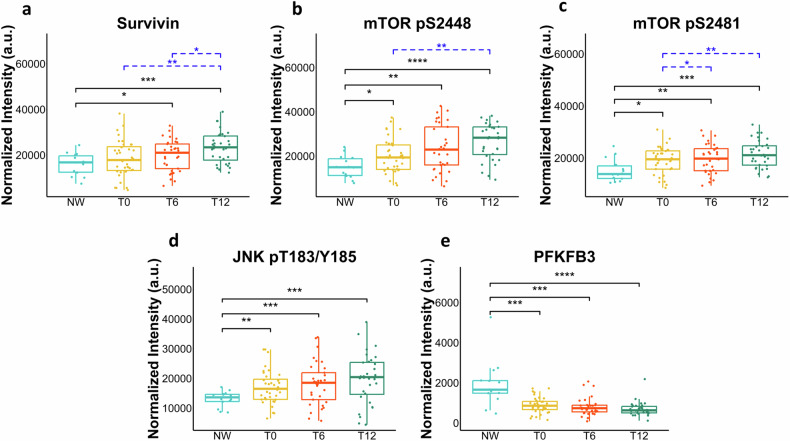
Metabolic redesign occurs in PBMC after bariatric surgery. Box plots resulting from the comparison of RPPA profiles of peripheral blood mononuclear cells (PBMCs) of severely obese patients before (T0) and six (T6), and 12 (T12) months after bariatric surgery and their normal-weight (NW) controls. **a**–**e** The plots represent the distribution of RPPA intensity values (mean ± SD), and statistical comparisons were performed using the Wilcoxon rank sum test for the comparison between OB and NW subjects. For the comparison at the different times, we used two tests for paired samples: Wilcoxon signed-rank test as non-parametric test and t-test as parametric test. Statistical significance is coded with an asterisk according to the level of significance (*p ≤ 0.05, **p < 0.01, ***p < 0.001, ****p < 0.0001).

As shown in Fig. 4, a trend towards increased levels of survivin (Fig. 4a) and a significant increase of phosphorylated mTOR (Fig. 4b, c) and JNK (Fig. 4d) were observed in pre-BS PBMCs of subjects with obesity versus the control counterpart. A further significant increase of survivin, mTOR Ser2448 and mTOR Ser2481 was observed at post-surgery times (Fig. 4a–c). Notably, in contrast to the findings in VAT, a significant decrease of PFKFB3 levels (Fig. 4e) was detected in PBMCs of individuals with obesity compared to the NW controls. In addition, PFKFB3 levels showed a decreasing trend in the post-surgery period, suggesting that a metabolic reconfiguration of glycolysis is occurring in post-BS PBMCs.

In conclusion, we show that pre-surgery PBMCs from individuals with obesity exhibit a tissue-specific RPPA profile with no alterations in the levels of DDR and senescence markers but changes in stress-response and metabolism markers when compared to NW controls. Conversely, a partial engagement of DDR and senescence pathways, together with the activation of proteins involved in cell adaptation to various stressors and metabolic changes, characterizes post-BS PBMCs.

### Mitochondrial health is restored in PBMCs after bariatric surgery

**Fig. 5:**
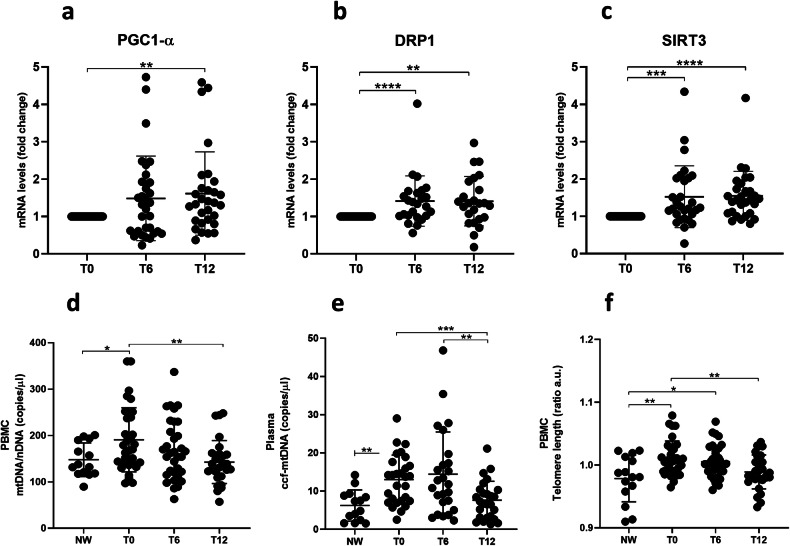
Bariatric surgery restores systemic mitochondrial homeostasis. Scatter plots resulting from the analysis of mitochondrial markers in whole blood, PBMCs and plasma samples by RT-PCR and ddPCR. Whole blood PGC1-α (**a**), DRP-1 (**b**) and SIRT3 (**c**) expression levels (mean ∓ SD) of severely obese patients before (T0) and six (T6) and twelve (T12) months after BS. Ratio between mitochondrial and nuclear DNA (mtDNA/nDNA) (mean ± SD) in PBMCs (**d**). Circulating cell-free mtDNA (ccf-mtDNA) levels in plasma samples before and after bariatric surgery (**e**). Telomere length (TL) by RT-PCR in PBMCs expressed in arbitrary units (A.U.) (**f**). Statistics were calculated using a two-tailed Student’s t-test (α = 0.05) if the requirements of normal distribution (Shapiro-Wilk test) were met. Otherwise, the Mann-Whitney test was performed. Non-parametric statistics (Wilcoxon signed-rank test) or paired t-tests were used to compare the levels of repeated measurements T0, T6, and T12. Statistical significance is coded with an asterisk according to the level of significance (*p ≤ 0.05, **p < 0.01, ***p < 0.001, ****p < 0.0001).

To gain a deeper understanding of the physiological consequences of this cellular response, we examined the expression levels of markers associated with mitochondrial health and function in the blood of obese patients, before and after undergoing BS. As shown in Fig. 5, we observed a significant increase in the mRNA levels of PGC1-α (Fig. 5a), Drp1 (Fig. 5b) and SIRT3 (Fig. 5c) in whole blood of BS patients at both the six- and twelve-months post-weight loss time points, suggesting an improvement in terms of mitochondrial quality and health. Moreover, a significant increase of mtDNA/nDNA ratio (Fig. 5d) was found in PBMCs of patients affected by obesity when compared to NW individuals. Interestingly, a gradual reduction in the mtDNA/nDNA ratio was observed after surgery. Remarkably, the mean value of this ratio, one year after surgery, was comparable to that observed in controls.

The presence of circulating cell free mitochondrial DNA (ccf-mtDNA) in the bloodstream is indicative of damaged mitochondria and inflammatory response [21]. We noticed a significant increase in ccf-mtDNA (Fig. 5e) levels in the plasma of subjects with obesity compared to NW individuals and, in addition, a progressive decrease in ccf-mtDNA levels was observed after BS, reaching the levels of control individuals. The trend of ccf-mtDNA in obesity and after BS reflects that of inflammatory markers, as previously reported [8] (Supplementary information Fig S1).

In summary, these findings suggest that the cellular response activated in PBMCs after BS is associated with the recovery of mitochondrial health. Ccf-mtDNA may serve as a promising marker for detecting mitochondrial dysfunction and systemic inflammation linked to obesity, both of which are mitigated following weight loss.

### Telomere length reverts to healthy range in PBMCs after bariatric surgery

Telomere dynamics and mitochondrial health are both susceptible to the impact of oxidative stress and inflammation, which are prominent characteristics of obesity. The analysis of telomere length (TL) (Fig. 5f) was performed in PBMCs from subjects with severe obesity before and after BS. Statistically significant lengthening of telomeres was observed in individuals with obesity at T0 compared to NW controls. To investigate whether this phenotype could be reversed when inflammation and oxidative stress were lowered, as occurs post BS [8], samples collected during the post-surgery time were analyzed. Statistically significant differences between patients affected by obesity and control individuals were still detected at T6, while at T12 after surgery, TL was comparable to that of NW individuals. Interestingly, these results are also paralleled by the recovery of mitochondrial health and the slowdown of inflammation, in agreement with the well-known functional relationship between telomeres and mitochondria.

## Discussion

In this study, we present the first evidence that chronic DDR and related stress response pathways are specifically activated in VAT but not in SAT in individuals with obesity. In obesity, VAT is characterized by hypertrophy, a key factor contributing to the release of inflammatory cytokines [22]. Consequently, VAT is more susceptible to inflammation and oxidative stress compared to the less metabolically active SAT. Our findings consistently reveal a VAT-specific activation of ATM and ATR along with downstream targets (i.e. H2AX, KAP1, Chk1, Chk2) suggesting that the accumulation of oxidative damage in this tissue triggers DDR activation. The pronounced activation of mTOR observed specifically in VAT is likely to play a significant role in influencing adipocyte function and metabolic health. Prolonged mTOR activation has indeed been shown to induce the conversion of brown adipocytes into a “white” state, which is linked to metabolic disturbances and obesity [23]. Distinct markers of VAT also include elevated levels of survivin and PFKFB3. Survivin, which is known to increase with obesity, acts as a protective mechanism, preventing apoptosis in adipose tissue stem cells and contributing to adipose tissue expansion [24]. mTOR signaling drives the increased expression of survivin in mature adipocytes from mice exposed to a high fat diet [25]. These findings align with our discovery of concomitant mTOR hyperactivation in VAT samples. Elevated expression of PFKFB3 in obesity has been previously reported [26]. We show increased expression of PFKFB3 in VAT, possibly supporting energy demands of DDR through increased glycolysis. Moreover, its contribution to oxidative stress induced double strand breaks repair has been recently documented [27]. Senescence serves as a protective mechanism triggered by DDR in the presence of prolonged or irreparable DNA damage [15]. Studies utilizing animal models have provided evidence supporting the causal involvement of senescence cells within adipose tissue in the development of metabolic dysfunctions associated with obesity [28]. In this study, we present evidence demonstrating that this phenomenon extends to adipose tissue in obese individuals. Concurrent with the activation of DDR, senescence markers p21 and p15-p16 are specifically activated in VAT. Furthermore, the mtDNA/nDNA ratio in VAT is higher than SAT, suggesting either an increase in mitochondrial biogenesis in response to increased energy demands or ongoing cellular stress potentially leading to mitochondrial dysfunction.

All these elements, coupled with the disruption of systemic adipokines and elevated levels of several pro-inflammatory cytokines observed in our study cohort [8], provide an example of how prolonged obesity in humans can lead to the development of a SASP within the primary target tissue.

A clear tissue-specificity emerged when the same markers were analyzed in the PBMCs. At the enrolment time, no significant differences in the levels of DDR and senescence markers were observed between obese and NW subjects, though a trend toward increased levels in obesity was noted. It should be considered that while adipocytes may accumulate DNA damage and senescent cells over time due to their relatively long lifespan (up to many years), PBMCs, because of their much shorter half-life, may show more dynamic and rapid responses. Increased levels of phosphorylated mTOR and JNK were observed in PBMCs of subjects with obesity as compared to NW subjects (Fig. 4a–d). Both are involved in the pro-inflammatory state and insulin resistance associated with obesity [29, 30]. Notably, in contrast to VAT, a significant decrease in PFKFB3 levels was detected in PBMCs of obese subjects, indicating a tissue-specific variation in the expression of this enzyme.

When the RPPA profile of our markers was evaluated post-surgery, a robust activation of ATM and ATR, along with increased level of KAP1, mTOR, JNK and survivin, were observed, persisting for up to 12 months. Some activation of H2AX and p15-p16 was also observed, while the levels of Chk1 and Chk2, as well as p21, did not change throughout the post-surgery period. ATM and ATR activation is a multifaceted process playing a role not only in DNA repair but also in regulating the cellular survival response to new challenges [31]. Based on our findings, we propose that, following BS-induced weight loss, PBMCs experience a set of adaptive mechanisms and processes to cope with the new metabolic conditions resulting from the weight loss, with the primary goal being to maintain cell viability. Essential components of the cellular survival response encompass recognition of stress, DNA repair, control of the cell cycle, anti-apoptotic processes, metabolic adaptation, activation of stress-responsive genes, immune reactions, epigenetic alterations, and autophagy, all working together to maintain cellular homeostasis. In the context of post-BS induced weight loss, these elements are all observable in PBMCs. Epigenetic changes and immune reactions are not addressed here but have been widely reported in several studies including our own conducted on the same cohort [32]. Several studies suggest ongoing DNA repair in PBMCs post-surgery, as indicated by the reduction of chromosomal damage [11, 12, 33]. The activation of ATM, ATR, and KAP1 that we observe over time may reflect activation of DDR and chromatin remodeling at DNA damaged sites. We did not observe a reduction of γ-H2AX levels post-BS as confirmed by two methodologies, RPPA and WB. This appears to contrast with the observed reduction of γ-H2AX foci in post-BS PBMCs. However, it is important to note that we measured the average levels of γ-H2AX in bulk samples rather than γ-H2AX foci, which specifically identify cells with double strand breaks. Additionally, cells can phosphorylate H2AX also in response to cellular stress beyond DNA damage [34]. Despite their usual quiescence, PBMCs have the capacity to transition between quiescent and active states in response to various signals. Such a transition has also been documented in PBMCs of bariatric patients following weight loss, showing a significant increase in their proliferation index and mitosis, coupled with a reduction in apoptosis that persists for up to 12 months after surgery [11]. This observed activation aligns with the increased levels in our post-surgery PBMCs of the stress-responsive proteins mTOR complexes and JNK as well as of survivin. Importantly, these elevated levels, known to promote cell proliferation, are sustained for up to 12 months post-surgery.

Conflicting results have been reported in the literature concerning TL changes in lymphocytes from obese subjects as well as after BS [35]. Our study revealed longer TL in lymphocytes from obese subjects compared to NW individuals. Post-surgery, TL returned to the control range. Telomere shortening may be linked to increased PBMCs proliferation after surgery [11]. Alternatively, the accumulation of oxidative base damage within telomeric sequences might be responsible for destabilization of telomeric G-quadruplex structures resulting in telomere lengthening [36, 37]. This process could explain both increased TL in obese subjects and the restoration of telomere structure post-surgery concomitantly with reduced systemic oxidative stress and inflammation as previously reported [8].

The adaptive changes are expected to impact energy metabolism to cope with the rapid weight loss. We show a reduction in PFKFB3 as a function of the post-surgery time, suggesting that a restructuring of glycolysis is occurring. Suppression of glycolysis has previously been observed in the presence of extensive DNA damage in association with an enhancement of the antioxidant response [38]. This occurs as cells prioritize DNA repair and reduce energy-demanding processes such as glycolysis.

To investigate whether this shift in metabolism is associated with an improvement of mitochondrial health, we conducted an analysis of indicators of mitochondrial function. PGC1-α, SIRT3, and Drp1 are interconnected components of the complex regulatory network that governs mitochondrial dynamics and energy metabolism. Their role in the development of metabolic syndromes has been suggested by findings in both knockout mice and humans [39]. A significant increase in the expression levels of proteins associated with mitochondrial dynamics and PGC1-α was found in the leukocytes of patients with obesity who underwent Roux-en-Y gastric bypass surgery at a one-year follow up [40]. Similarly, a significant increase in *pgc1*-α, *sirt3* and *drp1* gene expression levels was measured in whole blood of our bariatric patients after surgery.

Current evidence shows that the mitochondrial dysfunction in tissues and organs can be responsible for the release into the blood stream of several endogenous DAMPs able to trigger innate immunity [41]. Oxidized mt-DNA fragments escape mitochondria and are specifically responsible for the activation of the inflammasome [42]. The increase in ccf-mtDNA that we observed in the plasma of subjects with obesity and its trend toward decrease after BS testifies not only to the recovery of mitochondrial health and the prompt reversibility of this inflammatory index after weight loss, but also to its value as a predictive biomarker for metabolic syndrome development.

**Fig. 6:**
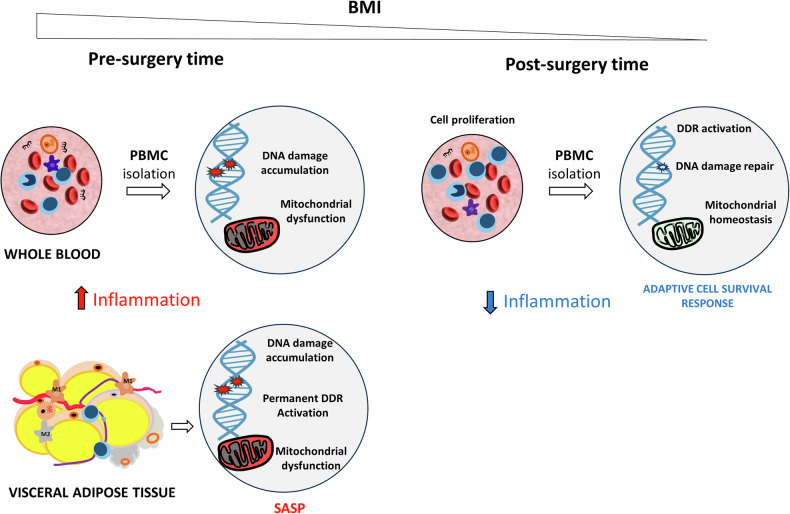
A hypothetical model: the dual role of DNA damage response in obesity. Under excessive caloric intake, hypertrophic expansion of white adipose tissue leads to adipocyte dysfunction, necrosis and fibrosis leading to low-grade systemic inflammation. At the molecular level, we speculate that DNA damage accumulation, chronic DDR activation, cellular senescence and mitochondrial dysfunction in visceral adipocytes concur to the release of autocrine and paracrine signals. These signals contribute to the development of a senescence-associated secretory phenotype (SASP), which is a systemic maladaptive homeostasis. Within the bloodstream, PBMCs in their quiescent state accumulate DNA damage without DDR activation. Following massive weight loss post-bariatric surgery, PBMCs shift toward an active state, characterized by cell proliferation, DDR activation associated with DNA repair and recovery of mitochondrial homeostasis. This adaptive cell survival response potentially contributes to the beneficial health effects of bariatric surgery. M1, M2: macrophages 1 and 2.

The main strength of this work is the longitudinal design of the study, allowing for repeated observations at an individual level. This approach effectively mitigates inter-individual variability, a potent confounding factor in human studies, thereby compensating for the relatively limited number of patients analyzed. Our results unveil distinctive metabolic patterns in specific tissues of obese subjects, such as adipose tissue and PBMCs. This suggests caution in utilizing markers from surrogate tissues. We identify a dual role of DDR and related stress response pathways, dependent on the context and cell type. These pathways may either be associated with a SASP in adipose tissue, contributing to metabolic syndrome in obese subjects, or they may support cell survival and DNA repair, promoting a healthier profile in circulating lymphocytes post- BS (Fig. 6). Further exploration is necessary to establish mechanistic connections among the analyzed proteins and to correlate proteomic profiles with clinical outcomes.

In summary, our findings support the notion that post-BS, an adaptive cellular response is triggered to accommodate the metabolic changes stemming from weight loss. This response may, at least in part, contribute to the positive health outcomes associated with BS.

## Materials and Methods

### Study design

A cohort of 36 subjects with severe obesity (33 females; mean age 47.1 ± 10.8 years; mean BMI 44.3 ± 6.9 kg/m^2^) attending the outpatient service of the Obesity Center of the University Hospital “Policlinico Tor Vergata” (Rome, Italy) for clinical evaluation before BS were enrolled and 32 patients (89%) completed the study. Eligible patients met the criteria for BS and had a stable body weight in the last 3 months preceding the evaluation. All patients are of Caucasian origin and lived in Italy. Exclusion criteria were: chronic liver or kidney disease; infections; malignancy; other acute or chronic systemic diseases; use of glucocorticoids, nonsteroidal anti-inflammatory medications, antibiotics, prebiotics, and probiotics intake in the last 3 months prior to BS. All patients underwent laparoscopic BS, mostly restrictive procedures (sleeve gastrectomy 17 [47.2%]; banded sleeve gastrectomy 13 [36.1%]; adjustable gastric banding 1 [2.8%]; banded Roux-en Y gastric bypass 3 [8.3%]; mini-gastric bypass 2 [5.6%]). At enrolment (T0) and 6 (T6) and 12 (T12) months after surgery, all the participants underwent a comprehensive medical evaluation, including anamnestic interview, physical examination, and the collection of blood samples for biomarkers measurements. After BS, patients received periodic counseling about dietary and lifestyle modifications as recommended [43]. At the end of the follow up, the BMI mean was 29.8 ± 5.1 kg/m^2^.

Anthropometric (weight, height, BMI, waist and hip circumference), clinical (blood pressure, heart rate) and hematological and biochemical parameters were assessed as previously described [44, 45]. Before BS, SAT and VAT biopsies and PBMCs were collected both before and after surgery (see Samples collection).

To obtain reference values for our biomarkers, we also enrolled 15 NW subjects (69% female; mean age 36.6 ± 11 years; mean BMI 23.5 ± 2 kg/m^2^) who underwent the same clinical assessment as bariatric patients only once. Among these, 15 NW subjects provided blood samples and 6 NW individuals who underwent elective laparoscopic surgery (cholecystectomy), provided samples of subcutaneous (NW-SAT) and omental adipose tissue (NW-VAT). All NW subjects met the same exclusion criteria adopted for bariatric patients. All the analysis has been carried out in a blinded manner. The study was approved by the ethical committees of Istituto Superiore di Sanità (prot. PRE 173116 of 15 March 2016; PRE-BIO-CE 10938 of 6 April 2018) and of the University Hospital ‘Policlinico Tor Vergata’ (protocol of the study connecting DNA 169/15) and all patients signed an informed consent. A partial set of data obtained in these two cohorts has been described in our recent publication [8].

### Samples collection

Blood samples were collected in the morning from fasting subjects and analyzed immediately (hematology and clinical biochemistry assays) or processed and stored in aliquots at −80 °C (whole blood, serum and plasma) until use.

PBMCs were isolated from whole blood within 2 h after collection using a standardized protocol designed to minimize sample processing time. Briefly, BD Vacutainers CPT™ (8 ml draw volume each), containing sodium citrate as anti-coagulant, were used and PBMCs and plasma were separated accordingly to the manufacturer instruction (on average 5 to 8 × 10^6^ cells/vacutainer were isolated). Moreover, for total RNA purification, 2.5 ml of whole blood was also collected into PAXgene® Blood RNA Tubes (PreAnalytix QIAGEN, Inc., Germantown, MD, USA) and cryopreserved at −80 °C. This methodology guarantees the stability of intracellular RNA for years. Furthermore, BD Vacutainer™ SST™ II Advance Tubes were used for the separation of the serum from the cellular components. Urine samples were also collected and cryopreserved. The SAT and VAT biopsies were cut in aliquots, immediately frozen and cryopreserved at −80 °C. Obtaining adipose tissue samples from NW subjects was limited (5-6 samples) due to difficulties in identifying subjects who met the exclusion criteria (see M&M). Nevertheless, the variability between individuals enabled us to achieve statistical significance.

### Reverse-phase protein microarrays

Reverse-Phase Protein microArrays (RPPA) analysis was performed following established protocols [46, 47]. Briefly, PBMCs lysis was performed using T-PER buffer (Thermo-Fisher Scientific) added with 60 μL/mL 5 M NaCl, 1X Protease Inhibitor cocktail, 1X Phosphatase Inhibitor Cocktail II and 1X Phosphatase Inhibitor Cocktail III (Sigma-Aldrich) for a maximum of 30’ on ice, followed by refrigerated centrifugation 10’ at 13.000 rpm. Supernatants (i.e. protein extracts) were collected, quantified using Bradford method (Bio-Rad Laboratories) [48] and stored at −80 °C. Lysis of adipose tissue was performed using the same protocol but samples underwent mechanical dissociation. Protein lysates were resuspended in Laemmli sample buffer [49] at a final concentration of 0.5 mg/mL with 2.5% TCEP reducing agent (Thermo-Fisher Scientific) and boiled for 3’ prior to printing with an Aushon 2470 (Quanterix) microarrayer equipped with 185 μm pins. RPPA samples were printed in technical triplicates onto nitrocellulose-coated slides (Grace Bio-labs). Series of control cell extracts (HeLa ± Pervanadate, Jurkat ± Etoposide, Jurkat ± Calyculin A, A431 ± Pervanadate and A431 ± EGF) were printed as 10%-fold-decrease mixtures of treated and untreated samples in a ten-point dilution curve format. Printed slides were promptly collected and stored at -20 °C for later use. Total protein content of printed slides was measured using Sypro Ruby (ThermoFisher Scientific). Immunostaining was performed by means of an automated system (DAKO AutostainerLink 48) using selected antibodies that had been pre-validated for RPPA and a commercially available signal amplification kit (Agilent/DAKO GenPoint). Prior to immunostaining, slides underwent an antigen retrieval (reblot) step (Millipore) followed by 2 hours blocking with 0.2% I-Block™ Protein-Based Blocking Reagent (Thermo-Fisher Scientific) in PBS. The tertiary reagent used for signal detection was streptavidin-conjugated IRDye680LT (LI-COR Biosciences). Stained slides were scanned by a Power Scanner (TECAN) and 16-bit images were analyzed via MicroVigene v5.2 software (VigeneTech) to detect spots, normalize signal and output RPPA data tables. The primary antibodies are listed: ATM (phospho S1981), Abcam ab81292; ATR (Ser428), Cell Signaling Technology 2853; Chk1 (Ser317), Cell Signaling Technology 12302; Chk2 (Thr68), Cell Signaling Technology 2661; Histone H2A.X (Ser139), Cell Signaling Technology 9718; KAP1 pS284, Bethyl Labs A300-767A; mTOR (Ser2448) (D9C2), Cell Signaling Technology 5536; mTOR (Ser2481), Cell Signaling Technology 2974; p15/p16 (C-7), Santa Cruz Biotechnology sc-377412; p21 Waf1/Cip1, Cell Signaling 2947; PFKFB3, Cell Signaling Technology 13123; SAPK/JNK (Thr183/Tyr185), Cell Signaling Technology 9251; Survivin (71G4B7E), Cell Signaling 2808. The original data of RPPA analysis are available in the section of supplementary materials (Table S2 and Table S3). Furthermore, we compared the results obtained in two independent experiments to check the reproducibility; linear regressions and Pearson’s correlation coefficient have been analyzed for pATM and γ-H2AX in 31 patients at the baseline (Supplementary Table S4). For a subset of patients, we performed a validation test of RPPA data by western blot analysis (Supplementary information Fig S2 and Fig S3).

### Droplet digital PCR and Real time PCR

Norgen genomic DNA isolation kit (NorgenBiotek Corp. cat. n. 24700) was used to isolate total DNA from PBMCs according to the manufacturer instructions. Relative mitochondrial DNA copy number was analyzed by digital droplet PCR (Biorad QX 200). Mitochondrial and nuclear DNA were detected by using ND2 and Rplp0 single tube Taqman real-time PCR assay, respectively (cat. n. 4331182; Life Technologies, Austin, TX, USA). Ccf-mtDNA levels were analyzed by digital droplet PCR in plasma samples. This analysis was carried out directly in 0.5 μl of plasma using the same PCR assays used in the real time PCR, without previous DNA isolation [50].

Total RNA from whole blood samples was extracted according to the manufacturer instructions (PAXgene blood RNA kit, PreAnalytix, Qiagen/BD company). Complementary DNA was retro-transcribed by the high-capacity cDNA reverse transcription kit (cat. no. 4368813; Life Technologies) and the gene expression analysis was carried out by quantitative real-time PCR (qRT-PCR). The gene expression levels of *sirt3*, *drp1* and *pgc1*-α, were calculated using specific Taqman assays (FAM labeled) by comparative method (2 ^–ΔΔCt^). rRNA 18 S assay (VIC labeled) was used as housekeeping reference gene. The original data are shown in supplementary materials (Tables S5, S6 and S7).

### Telomere length

Telomere length was measured by qRT-PCR using DNA samples isolated from PBMCs as described above. This method is based on the rationale that the amount of telomere signal per genome measured by qRT-PCR represents the average telomere length in a given DNA sample [51]. Telomere length was quantified as the relative ratio of telomere (T) repeat copy number to a single copy gene (S), called the T/S ratio, in experimental samples using standard curves. The Rplp0, encoding acidic ribosomal phosphoprotein P0, was used as the control single copy gene needed to quantify input genomic DNA and to normalize the signal from the telomere reaction. The primer sequences for telomere amplification were: TelF 5’-GGTTTTTGAGGGTGAGGGTGAGGGTGAGGGTGAGGGT-3’, TelR 5’-TCCCGACTATCCCTATC CCTATCCCTA TCCCTATCCCTA-3’. The primer sequences for the amplification of the reference gene Rplp0 were: Rplp0F 5’CAGCAAGTGGGAAGGTGTAATCC3’; Rplp0R 5’CCCATTCTATCATCAACGGGTACAA3’. The reaction was carried out in triplicate. The PCR master mix included 10 µl of SYBR Green PCR master mix (Bioline), 2 µl of forward primer and 2 µl of reverse primer (final concentration 100 nM), 5 µl (20 ng) of stock DNA (4 ng/µl) and purified water to a total volume of 20 µl. A standard curve and a negative control (no DNA template) were included in each experiment. For the standard curve, the reference DNA sample was diluted serially to produce six final concentrations (20, 10, 5, 2.5, 1.25 and 0.625 ng/µl). The PCR cycling condition for both amplicons were 95 °C for 20 s, followed by 40 cycles at 95 °C for 3 seconds and 60 °C for 30 s. The specificity of the PCR reaction was checked through the analysis of melting curves, obtained at the end of each PCR. The resulting T/S ratio represented the average telomere length per genome. To determine equal copy numbers per cell, the beta-globin gene was used as the housekeeping gene and amplified in all DNA samples. Experimental variability was corrected applying the Pfaffl model [52]. All PCRs were performed using the ABI Prism 7000 Sequence Detection System (Applied Biosystems). Fluorescence was analyzed with the ABI Prism 7000 SDS software (v2.0.5) to quantify PCR products for each sample based on the standard curve. The original data are shown in supplementary materials (Table S8).

### Statistical analysis

All analyses were conducted using R (v.4.3.0). Both parametric and non-parametric tests were performed to compare several DDR and metabolic markers between controls and patients with obesity before and after BS. Using the Shapiro-Wilk test we opted for either the t-test or the Wilcoxon test. Moreover, to compare patients before and after BS we used paired analysis, while to compare controls and patients with obesity we used unpaired analysis. Only the differences between groups with p value < 0.05 have been considered significant. Finally, to plot the results we used two R packages: ggplot2 (v.3.4.3) (https://ggplot2.tidyverse.org) and ggpubr (v0.6.0) (https://rpkgs.datanovia.com/ggpubr/). Statistical significance was coded using asterisk according to the level of significance (ns= not significative, *p < 0.05, **p < 0.01, ***p < 0.001, ****p < 0.0001). For gene expression and ccf-mtDNA, we used GraphPad Prism 8.0.2 for statistical analyses and graphs. When comparing two groups, a two-tailed Student’s t-test (α = 0.05) was performed if the requirements for normal distribution (Shapiro-Wilk test) and homogeneity of variances (F-test) were met. Otherwise, the Mann-Whitney U test was performed. Non-parametric statistics (Wilcoxon signed-rank test) or paired t-tests were used to compare the levels of repeated measurements (T0, T6, and T12). As previously described in [8], where we employed the same cohort of patients, we powered our study based on the expected change in micronuclei frequency in the blood mononuclear cells of patients upon BS, as DNA damage was the primary aim of the grants that funded the original research. The calculated sample size for obese was 22.3 patients per group; we instead recruited 50% more patients to obtain accurate results. Our sample size was calculated through a paired t-test power calculation with the R package pwr (pwr: Basic Functions for Power Analysis. R package version 1.3-0, https://cran.r-project.org/package=pwr) using a power of 0.95 and an effect size of 0.8. The longitudinal nature of the analysis (before and after bariatric surgery) can provide high accuracy when observing changes. For the control group, we estimated the need of at least 13 patients to perform a comparison with 36 obese patients to achieve a good power of 0.7. Considering that the number of control patients analyzed was relatively small, weaker associations could be missed. To this regard, it should be considered that this study had a main explorative nature aimed to detect interesting signals that may be worth of further investigation in subsequent better-powered studies.

## Supplementary information

Supplementary information

Original data Table S2-S3-S4-S5-S6-S7-S8

## Data Availability

The data that support the findings of this study are available from the corresponding author upon reasonable request.
