# Supplementary material for: The dual nature of DNA damage response in obesity and bariatric surgery-induced weight loss
Source: Cell Death Dis. 2024 Sep 11;15(9):664. doi: 10.1038/s41419-024-06922-0 (PMC11387396; doi:10.1038/s41419-024-06922-0)
Supplement: Supplementary file 1 — Supplementary information [file 41419_2024_6922_MOESM1_ESM.pdf]

Supplementary information

Characteristics	T0<br>Median (IQ range)	T6<br>Median (IQ range)	T12<br>Median (IQ range)	*p-value<br>T0 vs T6	*p-value<br>T0 vs T12	*p-value<br>T6 vs T12
**Number**	36 (3M/33F)	33 (2/31)	33 (2/31)			
**Weight (kg/m2)**	115.5 (103-130.5)	86.7 (77.3-96)	76.5 (68.05-91.6)	<0.0001	<0.0001	<0.0001
**BMI**	44.28 (39.74-48.32)	33.45 (30.6034.80)	29.81 (27.10-32.21)	<0.0001	<0.0001	<0.0001
**Waist circumference (cm)**	122 (114-129)	100 (90-107)	93.25 (83.75-101.5)	<0.0001	<0.0001	<0.0001
**Hips circumference (cm)**	141 (132-150)	120 (110-127)	110.5 (103.75-119)	<0.0001	<0.0001	<0.0001
**W-H ratio**	0.86 (0.83-0.91)	0.83 (0.80-0.89)	0.83 (0.79-0.88)	0.0316	0.0058	0.0997
**Current smoker (%)**	16.6	16.6	14.28	>0.999	>0.999	>0.999
**Glycemia mg/dl**	93 (87-102.5)	81 (77-87)	85 (80-91.5)	<0.0001	<0.0001	0.0636
**Insulin (µU/ml)**	17.95 (14.78-22.13)	7.8 (6.1-8.7)	7.4 (5.4-9.6)	<0.0001	<0.0001	0.4404
**HOMAIR**	4.32 (3.00-5.21)	1.51 (1.17-1.98)	1.50 (1.05-2.07)	<0.0001	<0.0001	0.5052
**HbA1c**	38 (36-43)	34 (32-37)	34 (32-36)	<0.0001	<0.0001	0.5831
**Total cholesterol (mg/dl)**	200.5 (168.5-224.5)	195 (163-230)	202 (176.5-234.5)	0.5857	0.1635	0.0444
**HDL-C (mg/dl)**	50 (41-57)	55 (47-62)	55.5 (49.5-67)	0.0293	0.0013	0.0016
**LDL-C (mg/dl)**	123.6 (95.8-148)	120.4 (96.8-147.2)	122.6 (105.3-154.8	0.8094	0.5434	0.5435
**Triglycerides (mg/dl)**	124 (96-179)	95 (73-126)	84.5 (75-110.5)	<0.0001	<0.0001	0.0937
**ALT (U/l)**	29 (22.5-38.5)	18 (14-22)	18 (13-21.5)	0.0002	0.0007	0.3340
**AST (U/l)**	20 (16-24)	18 (16-21)	20 (15-24.5)	0.2517	0.4539	0.1229

Table S1. Clinical and biochemical profiles of the human cohort. Median values and interquartile ranges of studied variables measured in obese patients before (T0) and 6 months (T6) and 12 months (T12) after bariatric surgery. The p-value for difference between T0, T6 and T12 groups were calculated by Wilcoxon rank-sum test.

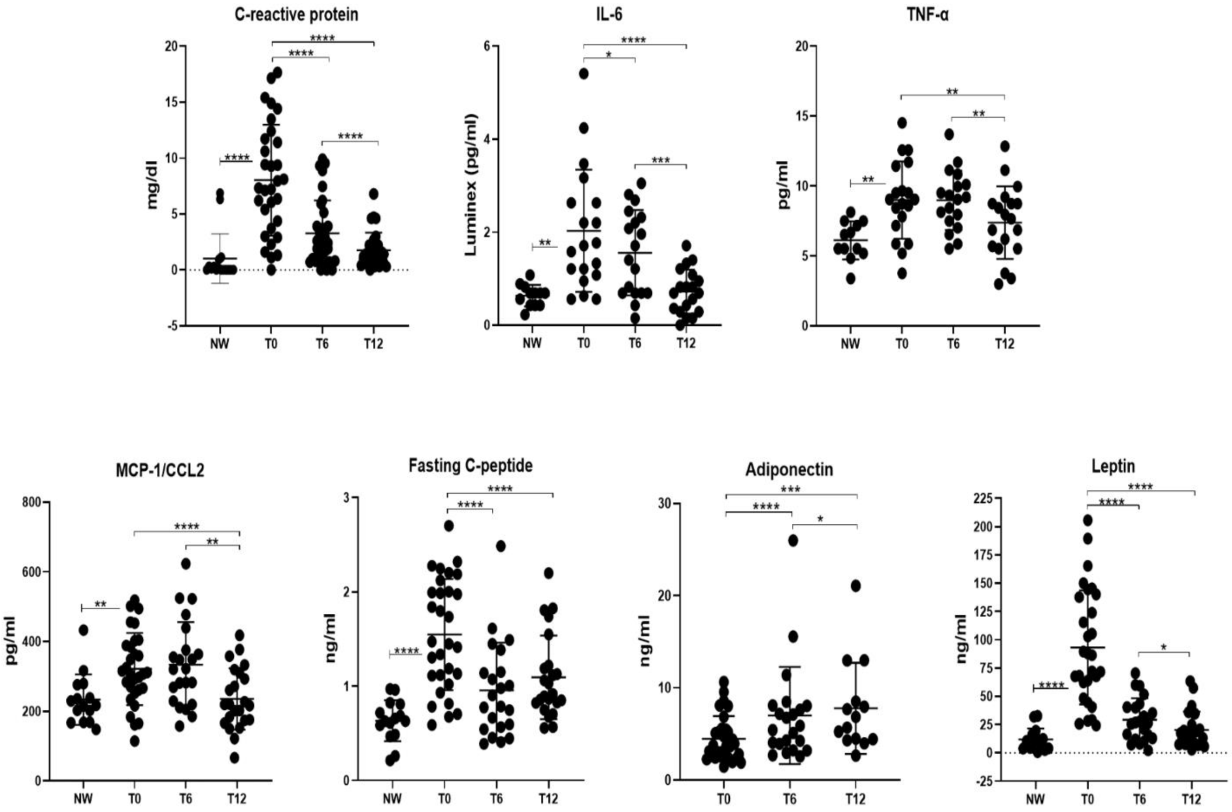

Fig S1. Analysis of inflammation markers in patients affected by obesity before and after surgery. Mean values with SD are reported for enrolled severely obese patients before (T0) and six (T6), and 12 months (T12) after bariatric surgery. A cohort of normal-weight (NW) subjects was also analysed as a reference. A two-tailed Student's t-test ($\alpha = 0.05$) was performed if the requirements of normal distribution (Shapiro-Wilk test) were met. Otherwise, the Mann-Whitney test was performed. Non-parametric statistics (Wilcoxon signed-rank test) or paired t-tests were used to compare the levels of repeated measurements T0, T1, and T2 (b). The following indicators were used for all statistical tests: * $p < 0.05$; ** $p < 0.01$; *** $p < 0.001$; and **** $p < 0.0001$.

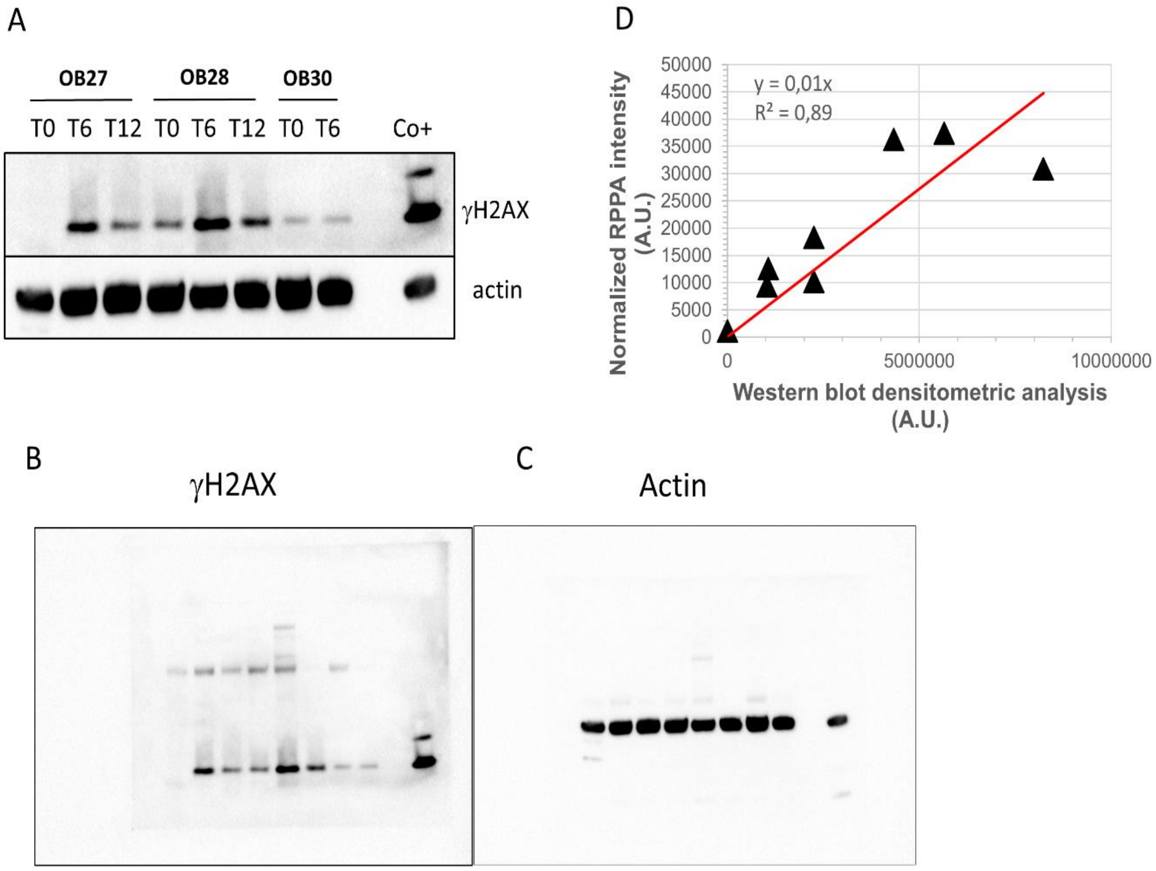

Fig S2. Original western blot for validation of RPPA. (A) Validation of γH2AX RPPA results by western blot analysis for a set of PBMC samples from obese subjects (OB27, OB28, OB30) at the enrolment time (T0); six months after BS (T6); one year after BS (T12). A complete picture of the western blot analysis is shown (A). 4Gy irradiated human primary fibroblasts were used as positive control (Co+). Full and uncropped western blot filters for gH2AX (B) and actin (C) are shown. "Intensity values (A.U.) obtained from RPPA and densitometric analysis of western blotting for H2AX activation are plotted along with the corresponding equation and coefficient of determination (R2) of the linear regression (D). Pearson's correlation coefficient: 0,85.

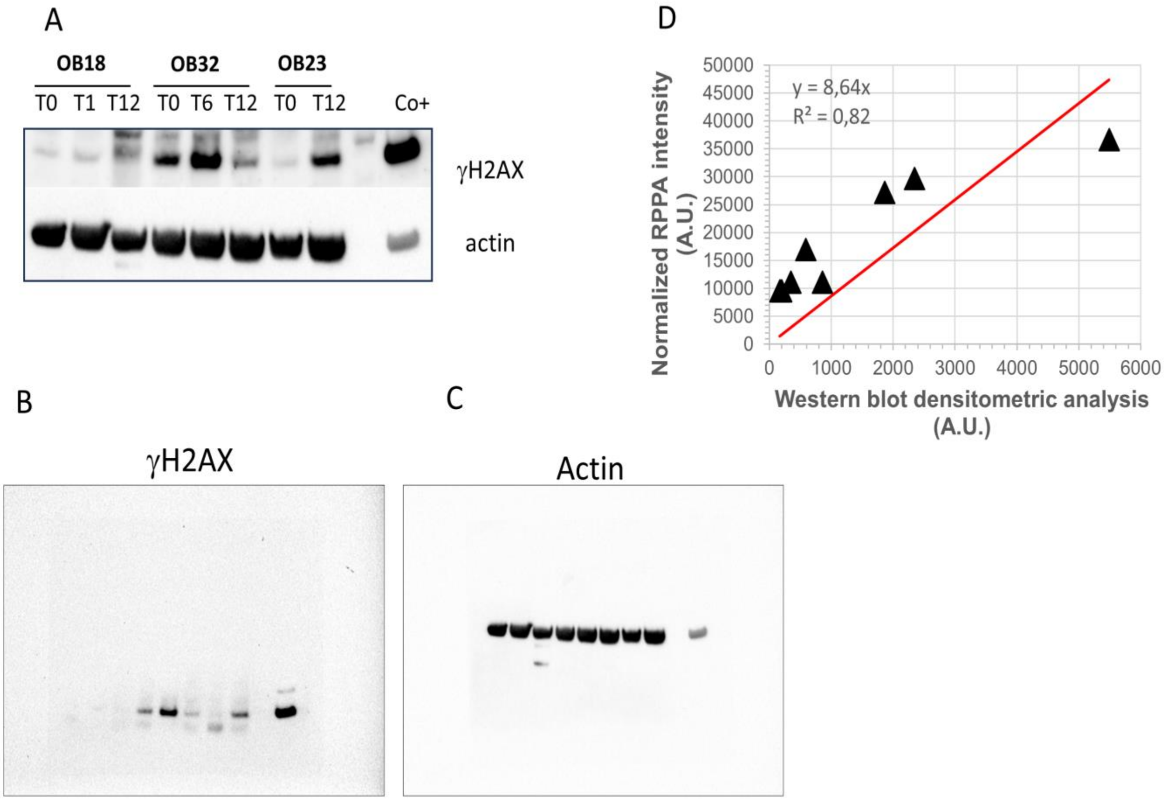

Fig S3. Original western blot for validation of RPPA. (A) Validation of γH2AX RPPA results by western blot analysis for a set of PBMC samples from obese subjects (OB18, OB32, OB23) at the enrolment time (T0), six months after BS (T6) and one year after BS (T12). A complete picture of the western blot analysis is shown (A). 4Gy irradiated human primary fibroblasts were used as positive control (Co+). Full and uncropped western blot filters for γH2AX (B) and actin (C) are shown. "Intensity values (A.U.) obtained from RPPA and densitometric analysis of western blotting for H2AX activation are plotted along with the corresponding equation and coefficient of determination (R2) of the linear regression (D). Pearson's correlation coefficient: 0,91. Due to low levels of activated H2AX in part of the samples, the densitometric analysis values for the second exemplificative western blot image (dashed arrow) were obtained after probing the filter with the primary antibody, followed by labelled streptavidin-biotin (LSAB) method for signal amplification. The LSAB method with subsequent chemiluminescent detection, allowed us to determine the levels of activated H2AX in sample with poor expression while leaving the relative sample intensities unaltered.
